# Epidemiological Evidence That Garden Birds Are a Source of Human Salmonellosis in England and Wales

**DOI:** 10.1371/journal.pone.0088968

**Published:** 2014-02-26

**Authors:** Becki Lawson, Elizabeth de Pinna, Robert A. Horton, Shaheed K. Macgregor, Shinto K. John, Julian Chantrey, J. Paul Duff, James K. Kirkwood, Victor R. Simpson, Robert A. Robinson, John Wain, Andrew A. Cunningham

**Affiliations:** 1 Institute of Zoology, Zoological Society of London, London, United Kingdom; 2 Salmonella Reference Service, Public Health England, London, United Kingdom; 3 Department of Bacteriology, Animal Health and Veterinary Laboratories Agency, Addlestone, Surrey, United Kingdom; 4 Veterinary Department, Zoological Society of London, London, United Kingdom; 5 School of Veterinary Sciences, University of Liverpool, Neston, Cheshire, United Kingdom; 6 Diseases of Wildlife Scheme, Animal Health and Veterinary Laboratories Agency, Penrith, Cumbria, United Kingdom; 7 Universities Federation for Animal Welfare, Wheathampstead, Hertfordshire, United Kingdom; 8 Wildlife Veterinary Investigation Centre, Truro, Cornwall, United Kingdom; 9 British Trust for Ornithology, Thetford, Norfolk, United Kingdom; 10 University of East Anglia, Norwich, Norfolk, United Kingdom; Institut National de la Recherche Agronomique, France

## Abstract

The importance of wild bird populations as a reservoir of zoonotic pathogens is well established. Salmonellosis is a frequently diagnosed infectious cause of mortality of garden birds in England and Wales, predominantly caused by *Salmonella enterica* subspecies *enterica* serovar Typhimurium definitive phage types 40, 56(v) and 160. In Britain, these phage types are considered highly host-adapted with a high degree of genetic similarity amongst isolates, and in some instances are clonal. Pulsed field gel electrophoresis, however, demonstrated minimal variation amongst matched DT40 and DT56(v) isolates derived from passerine and human incidents of salmonellosis across England in 2000–2007. Also, during the period 1993–2012, similar temporal and spatial trends of infection with these *S*. Typhimurium phage types occurred in both the British garden bird and human populations; 1.6% of all *S.* Typhimurium (0.2% of all *Salmonella*) isolates from humans in England and Wales over the period 2000–2010. These findings support the hypothesis that garden birds act as the primary reservoir of infection for these zoonotic bacteria. Most passerine salmonellosis outbreaks identified occurred at and around feeding stations, which are likely sites of public exposure to sick or dead garden birds and their faeces. We, therefore, advise the public to practise routine personal hygiene measures when feeding wild birds and especially when handling sick wild birds.

## Introduction

*Salmonella enterica* subspecies *enterica* serovar Typhimurium (*S*. Typhimurium) [1] is the bacterium responsible for passerine salmonellosis and this disease has been documented internationally as a cause of wild bird mortality since the 1950s, principally at garden bird feeding stations [2]–[4]. In humans, salmonellosis is caused by infection with any one of over 2500 *S. enterica* serovars, as classified by the Kauffmann-White scheme [5], and can result in serious disease with infant, geriatric and immunocompromised individuals being particularly vulnerable [6]. In Britain, *S.* Typhimurium is one of the commonest *Salmonella* serovars isolated from humans and from common garden birds such as greenfinches *Chloris chloris* and house sparrows *Passer domesticus.* These species are most frequently affected by salmonellosis, although other gregarious and granivorous species (e.g. bullfinch *Pyrrhula pyrrhula*, chaffinch *Fringilla coelebs*, goldfinch *Carduelis carduelis* and siskin *Carduelis spinus*) are also susceptible [7], [8]. The role of wild birds as reservoirs of zoonotic pathogens is an important research topic [9] as the likelihood of human contact with wild birds and their faeces is increasing with the large-scale provisioning of supplementary food for birds in private gardens [10] that has increased markedly over the last two decades.

Sub-typing of *Salmonella* has for years depended on phage typing and typical bird phage types can be found in humans. *Salmonella* Typhimurium definitive phage type (DT) 40, DT56 variant(v) and DT160 account for the majority of isolates from garden birds in Great Britain [8], [11]–[14]. Garden bird *S*. Typhimurium phage types are thought to have a narrow host range and may be highly host-adapted [15] and clonal in some instances [16]. A pilot study to characterise 32 passerine *S*. Typhimurium isolates from the north of England 2005–2006 found, on the basis of phage typing and pulsed-field gel electrophoresis (PFGE), that they were closely related and were fully sensitive to the panel of antimicrobials tested [17]. A study of 124 *S*. Typhimurium isolates derived from passerines from across England and Wales, 1997–2007, of which *S*. Typhimurium DT40 and DT56(v) accounted for 97% (120/124) of the isolates, found that PFGE groupings closely correlated with phage type with remarkably few exceptions, and that a high degree of genetic similarity (>90%) was observed within and between the two most common PFGE groupings [18]. Whilst *S*. Typhimurium isolates associated with wild birds (comprising DT40 and DT56(v) from passerines, DT41 and DT195 from gulls [Laridae spp.], DT2 and DT99 from feral pigeons [*Columbia livia*]) have been isolated from a range of livestock species, they accounted for less than 0.5% of *Salmonella* spp. recovered from cattle, sheep, pigs, chickens or turkeys in Great Britain, 1995–2003, and only 3% from extensively-reared birds (e.g. game, ducks and geese) [19]. Free-living wild animals (n = 5494), comprising 16 mammal species and 30 bird species (including 77 raptors, 62 waterfowl, 25 songbirds with no Fringillidae species and 19 corvids) were screened for *Salmonella* spp. carriage in Cornwall over the period 1974 to 1988; *S*. Typhimurium was isolated only from the badger *Meles meles* (0.7% infected, 34/4881 individuals) and no garden bird phage types (DT40, DT56(v) or DT160) were found [20]. Salmonellosis due to *S*. Typhimurium DT40 or DT56(v) has been described in domestic cats, but these are thought to have become infected through predation of passerine prey [21], [22]. Collectively, these findings support the hypothesis that wild passerine birds are the primary reservoir of these *S*. Typhimurium phage types.

Garden bird phage types of *S*. Typhimurium have zoonotic potential with infection via the faeco-oral route. Where epidemic mortality due to salmonellosis has occurred in garden birds, for example in Sweden [23] and New Zealand [16], humans also have become infected, with resulting enteric disease. Identical genotypes of *S*. Typhimurium in sympatric populations of wild birds and humans were demonstrated in Germany over a two-year period; direct or indirect pathogen transmission from wild birds to humans was proposed [24]. Similarly, house sparrow access to kitchen facilities with sporadic faecal contamination of food was postulated to be the origin of two outbreaks of gastroenteritis (due to *S*. Typhimurium DT40 and DT160) in hospital patients in Britain reported in the 1970s [12].

No national longitudinal studies to evaluate the zoonotic risk of garden bird-associated *Salmonella* spp. have been conducted to date and the extent to which wild birds act as a source of human infection is unknown. In this study, we used PFGE to investigate whether the most common *S.* Typhimurium phage types reported in garden birds in Britain in recent years (DT40 and DT56(v)) were the same strains isolated from humans. Additionally, spatio-temporal trends of *S*. Typhimurium DT40, DT56(v) and DT160 infection in garden birds and human beings in England and Wales from 1993 to 2012 were explored.

## Materials and Methods

### Ethics statement

No live animals were used for this research; however, the Garden Bird Health *initiative* (Project WLE/0460) was reviewed and approved by the Zoological Society of London's Ethics Committee.

Data on human incidents of infection were obtained from databases held by the Gastrointestinal Bacteria Reference Unit, Public Health England. All data were anonymized before use in this study in accordance with the Data Protection Act (UK) 1998 [25]. No humans were directly involved in this study. Anonymized data from this study is available to researchers on request.

### Garden bird mortality incidents with *S*. Typhimurium DT40, DT56(v) and DT160

From 1993 to 2005 and 2009 to 2012, opportunistic reports of garden bird mortality incidents in England and Wales were solicited from members of the public through a passive surveillance network, including the British Trust for Ornithology (BTO), the Royal Society for the Protection of Birds (RSPB), the Universities Federation for Animal Welfare and the Institute of Zoology (IoZ, [8]). From 2005–2008, a research project, entitled the Garden Bird Health *initiative* (GBH*i*), utilised a combination of opportunistic reports of garden bird mortality through a passive surveillance network, as above, in combination with complementary, but independent, systematic surveillance [26]. A network of veterinary investigation centres participated in the GBH*i*, co-ordinated by the IoZ, including the University of Liverpool and the Wildlife Veterinary Investigation Centre. Hereafter, for the purposes of this study, post-mortem examinations (PME) undertaken at the IoZ and GBH*i* participating laboratories will be referred to as the IoZ dataset.

Carcasses submitted for PME were examined following a standardised protocol as described in [8]. Details of the species, date found and geographical location (to the nearest 1 km using Ordnance Survey National Grid Reference) were recorded. Systematic external and internal examinations of body systems were performed and any gross lesions described.

The liver, intestine and/or crop/oesophagus, in addition to any gross lesions found, were routinely sampled and examined for the presence of pathogenic bacteria using a standardised protocol including *Salmonella*-selective enrichment media [8]. Bacterial isolates were identified using colony morphology and Gram's staining coupled with biochemical properties, which were determined using the analytical profile index (API) 20 Enterobacteriaceae biochemical test strip method (API-BioMerieux, Marcy l'Etoile, France). Slide agglutination tests were performed for the identification of suspected *Salmonella* spp. isolates using poly-O antisera (Pro-lab diagnostics, Neston, UK). Salmonella isolates were placed onto microbank beads (Pro-lab diagnostics) and stored at both −25°C and −70°C. All archived isolates were grown in pure culture from a single colony. Batches of isolates were submitted to the Animal Health and Veterinary Laboratories Agency (AHVLA) (1993–1998) and to the Salmonella Reference Service (SRS) (1999–2012) of Public Health England (formerly the Health Protection Agency) (PHE) for biotyping (serotype and phage type) according to standardised international protocols [27]. Antibiotic sensitivity testing was performed on a subset of the garden bird *S*. Typhimurium isolates at the SRS according to a standardised protocol [28].

A diagnosis of salmonellosis was made from birds where a *Salmonella* sp. was isolated from: 1. one or more gross lesions (oesophageal ulcers, focal abscesses or granulomata in soft tissues), or 2. from cases with hepatomegaly or splenomegaly, in the absence of any other obvious cause of death - each of these gross abnormalities are characteristic of salmonellosis [26]. A salmonellosis incident was classified as one or more dead birds found at a particular site within a 30-day period. Garden bird mortality incidents where a *Salmonella* sp. was isolated from a bird, with no gross abnormalities, were also collated.

Additionally, dead wild birds were submitted to AHVLA laboratories across England and Wales from 1993 to 2012. From 1998 to 2012, the submissions were part of the national network of the AHVLA Diseases of Wildlife Scheme (DoWS). Post mortem and microbiological examinations and phage typing were performed with minor modifications to the above protocols. The AHVLA database of *S*. Typhimurium DT40, DT56(v) and DT160 isolates from garden birds was reviewed to determine the number of mortality incidents for each phage type by year and county across the study period. IoZ and AHVLA datasets were combined for the analyses in order to provide the most comprehensive coverage possible. Both the IoZ and AHVLA datasets were the outcome of national schemes, with approximately equal surveillance effort across national government office regions (GORs). Duplicate incident reports from the period 1993–1998, when biotyping of IoZ isolates was performed by AHVLA, were removed from the dataset.

### Incidents of human infection with *S*. Typhimurium DT40, DT56(v) and DT160

Salmonella isolates from clinically ill patients were submitted by hospitals and other medical referral facilities to the SRS for serotyping and phage typing [27]. Human incidents of infection with *S*. Typhimurium DT40, DT56(v) or DT160 from 1993 to 2012 were reviewed. Isolates from people with a recent history of international travel and *S*. Typhimurium DT56(v) isolates with a multi-drug resistant profile characteristic of those frequently isolated in sub-Saharan Africa were excluded from the study [29], [30]. No information on the clinical signs at presentation was available although enteric infection was assumed to be the major presenting complaint. The sample from which the bacterium was cultured (i.e. blood, stool, other) from each patient was noted. Available patient histories and the pattern of PHE submissions were reviewed to determine whether each incident was considered a sporadic infection or part of a larger disease outbreak. Based on the available information, repeat samples from individuals and subsequent cases of family members, presumed to be infected through close contact rather than common exposure, were excluded from the study. Affected individuals were categorised by age as infant (0–5 years), child (>5–16 years), adult (>16–65 years) and geriatric (>65 years). The home town for each human incident was not available; instead the location of the referring medical facility, summarised by GOR, was used as an approximation to examine the incident distribution.

### Pulsed field gel electrophoresis comparison of garden bird and human-derived *S*. Typhimurium DT40 and DT56(v) isolates

Twenty-five *S*. Typhimurium human-derived isolates of both DT40 and DT56(v) were selected from the PHE archive; 2–5 isolates of each phage type were selected for each year, 2000–2007, from separate sites with a wide geographical distribution across England. Garden bird-derived *S*. Typhimurium isolates were selected from the available IoZ and AHVLA archives over the same time period (12 DT40 and 38 DT56(v)); where possible isolates were chosen in the same GOR as the human-derived isolates for that year, however human and garden bird-derived isolates were not knowingly available from the same or closely-located sites. No garden bird-derived *S*. Typhimurium DT160 isolates were available for PFGE.

Preparation of bacterial cell suspensions, agarose plugs and PFGE were performed according to the PulseNet USA *Salmonella* method [31]. All PFGE in this study was performed in the same AHVLA laboratory with the same equipment enabling robust, direct comparison between isolates. In summary, DNA from *Salmonella* isolates was digested for 2 hours at 37°C using 50 U *Xba*I endonuclease per sample (Promega, Southampton, UK). Macro-restriction digested fragments were separated on a 1% agarose gel (SeaKem Gold, LONZA, UK) at 6 volts per centimetre for 17.5 hours at 14°C on a CHEF DRIII system (Bio-Rad Laboratories). Pulse times were ramped from 2.2–63.8 seconds and a reorientation angle of 120° was applied. *Salmonella* serovar Braenderup H9812 was used as a control for normalisation. Gels were stained for 20 minutes in 1 litre of ethidium bromide solution (0.8 µg/ml) and then destained for 2 hours in deionised water (6×20 minutes with fresh water). Images were captured as tif files using an Alpha Imager 2200 (Alpha Innotech Corporation, USA) and UV light. BioNumerics version 3.0 (Applied Maths BVBA, Kortrijk, Belgium) software was used for image analysis. A percentage similarity between PFGE banding patterns was computed according to the Dice similarity coefficient method with a 1% tolerance window, and a dendrogram was constructed using the unweighted pair group method with averages.

### Data analyses

Comparisons were made between the frequencies of human incidents for each of the *S*. Typhimurium phage types by age group using the Pearson chi-square test. The Spearman rank correlation for non-parametric data was used to assess the association between the number of garden bird and human incidents for each *S*. Typhimurium phage type by year, 1993–2012, and the association between the spatial distribution of garden bird and human incidents for each *S*. Typhimurium phage type by GOR. The number of human incidents was stratified by the regional human population size (based on the 2001 national census, [32]) in order to correct, as far as possible, for variation in the human population density across the GORs. The absolute number of garden bird incidents was used in this analysis; since it was not possible to compile a meaningful relative abundance score for the multiple wild bird species affected by salmonellosis in the study. Both the greenfinch and house sparrow have widespread populations across England and Wales [33].

Statistical analyses were performed using R 2.15.2 [34] and statistical significance was given at P≤0.05. Spatial data were presented using ArcView 3.0 geographical information system (GIS) software (Environmental Systems Research Institute GIS and Mapping Software, California, U.S.A.).

## Results

### Garden bird incidents

During the period 1993–2012 inclusive, garden bird mortality incidents from which one of the three major garden bird-associated *S*. Typhimurium phage types (DT40, DT56(v) or DT160) were isolated were confirmed at 438 sites (218 incidents in the IoZ dataset and 220 in the AHVLA dataset). These comprised 32% DT40 (82 incidents in IoZ dataset and 59 in AHVLA dataset), 65% DT56(v) (129 incidents in IoZ dataset and 156 in AHVLA dataset) and 3% DT160 (7 incidents in IoZ dataset and 5 in AHVLA dataset). For the IoZ dataset, the majority of incidents were due to salmonellosis where the disease was considered the likely cause of death (98%, 213/218). For the remaining five incidents, the birds may have been carriers of *Salmonella* sp., or in an early stage of clinical disease, or have died of clinical disease with no gross abnormalities. The state of carcass preservation precluded histopathological examination of birds from many of these incidents to differentiate clinical disease and bacterial carriage. Post-mortem examination data were not available for review from the AHVLA incidents; however, as with the IoZ dataset, the vast majority of garden bird mortality incidents associated with these phage types was considered due to salmonellosis (P. Duff, *unpublished observations*).

Greenfinch and house sparrow were the species most frequently examined from the incidents in the IoZ dataset, comprising 60% (154/258) and 19% (49/258) respectively, with the remaining 21% from a further nine species (siskin n = 23, goldfinch n = 12, chaffinch n = 8, bullfinch n = 7, and brambling [*Fringilla montifringilla*], collared dove [*Streptopelia decaocto*], reed bunting [*Emberiza schoeniclus*], tree sparrow [*Passer montanus*] and wood pigeon [*Columba palumbus*] all n = 1). The two columbiforms were submitted from sites with concurrent confirmed passerine salmonellosis and neither bird had gross abnormalities consistent with salmonellosis. Similarly, garden bird incidents in the AHVLA dataset involved “finches” in 79% (173/220) of incidents and “sparrows” in 19% (42/220); species identification beyond bird group was not available for all cases but greenfinch was the most frequently identified species (45%; 99/220 incidents).

For the bird carcasses in the IoZ dataset, 95% (218/230) of the incidents from which *S*. Typhimurium isolates were subtyped belonged to the three study phage types. The remaining incidents consisted of six additional phage types that were isolated from single birds or mortality incidents (DT37, DT81, DT87v, DT120, U313, PT277), two incidents with *S*. Typhimurium DT1 and four incidents where the isolates were classified as “reacts does not conform”. Equivalent figures were unavailable from the AHVLA dataset, but the same three phage types similarly accounted for the vast majority of passerine-derived *S*. Typhimurium isolates (P. Duff, *unpublished observations*).

Antibiogram data were available from 90 passerine-derived *S*. Typhimurium DT40 and 151 DT56(v) isolates examined at the PHE from 2002–2011; all were fully sensitive to the panel of antimicrobials tested (amikacin, ampicillin, cephalexin, cephradine, cefotaxime, ceftriaxone, cefuroxime, chloramphenicol, ciprofloxacin, furaxolidone, gentamycin, kanamycin, nalidixic acid, neomycin, spectinomycin, tetracycline and trimethoprim).

### Human incidents

A total of 518 incidents of *S*. Typhimurium DT40 (n = 177), DT56(v) (n = 237) and DT160 (n = 104) phage types, were confirmed in humans from 1993–2012. Isolates were recovered from faecal samples with the single exception of a DT56(v) isolate recovered from blood culture from an adult in 2003. All incidents were considered sporadic infections and not part of larger disease outbreaks. Nearly half (47%) of reported human cases of infection with *S*. Typhimurium garden bird-associated phage types (DT40, DT56(v) and DT160 combined) came from infants (242/518), with the remainder being 13% children (66/518), 27% adults (142/518), 9% geriatric (47/518); in 4% of cases age was unknown (21/518). There was no significant difference in the demographic breakdown of human cases amongst the three *S.* Typhimurium phage types (χ^2^ = 10.11, df = 6, P>0.05).

### Pulsed-field gel electrophoresis comparison of garden bird and human-derived *S*. Typhimurium DT40 and DT56(v) isolates

**Figure 1:**
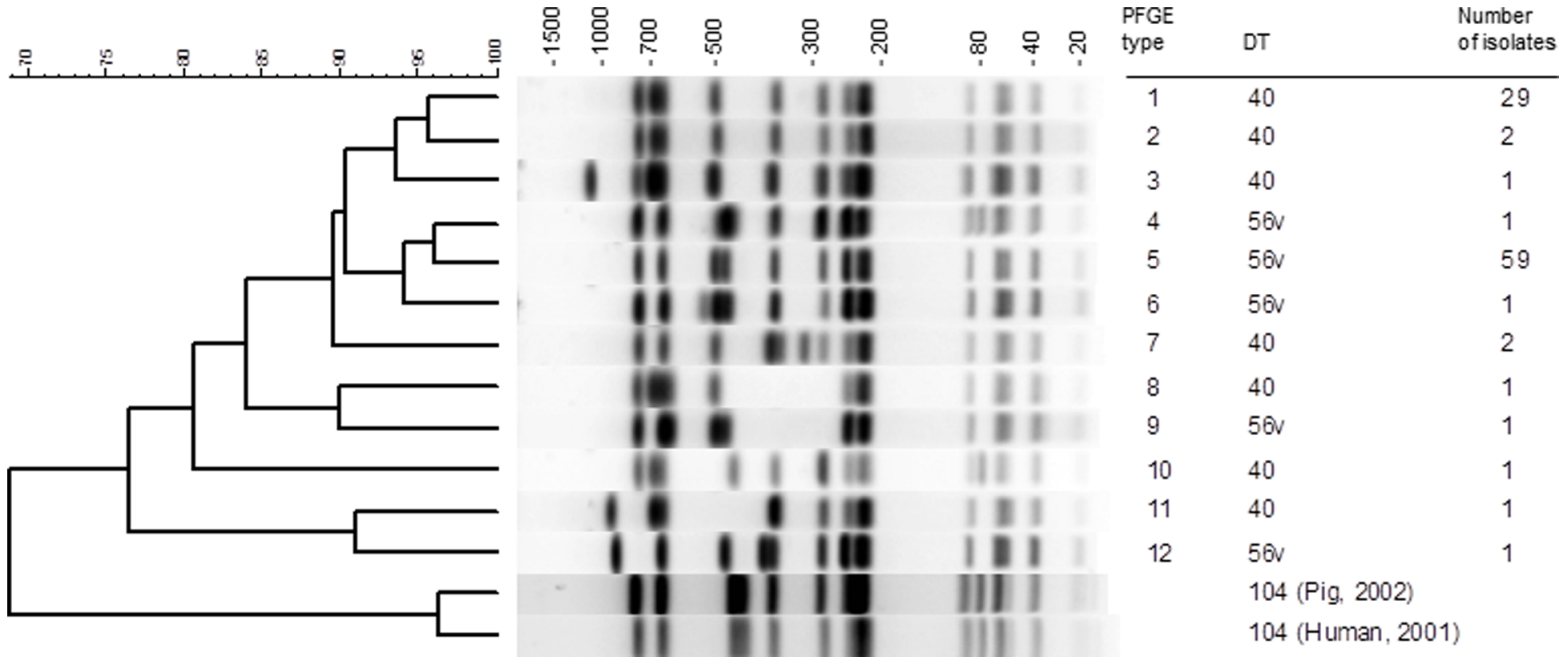
Pulsed-field gel electrophoresis on human and passerine-derived *Salmonella* Typhimurium DT40 and DT56(v) isolates. Dendrogram showing the percent similarity between representative patterns from *Salmonella* Typhimurium DT40 and DT56(v) isolates digested with *Xba*I restriction enzyme. PFGE band profiles are shown against kb scale. Phage type (DT40 and DT56v) and PFGE groupings (1–12) data are given along with the number of study isolates in each group. PFGE profiles of contemporary *S*. Typhimurium DT104 isolates from a human and a pig are included for comparison.

One hundred *S*. Typhimurium isolates collected from 2000–2007 inclusive were analysed using PFGE; these comprised 50 human-derived isolates (25 DT40 and 25 DT56(v)) and 50 garden bird-derived isolates (12 DT40 and 38 DT56(v)). Twelve unique banding profiles with an overall percentage similarity of 76.5% were classified as separate PFGE groups (Figure 1). Although each PFGE group consisted of a single phage type, there was no good association between the clustering of PFGE group and phage type. Eighty-eight percent (88/100) of isolates were in two of the PFGE groups (1 and 5) (Table 1). PFGE group 1 was comprised of 78% (29/37) of the *S*. Typhimurium DT40 isolates: 20/25 human and 9/12 bird-derived isolates, and clustered with groups 2 and 3 (Figure 1). PFGE group 5 was comprised of 94% (59/63) of the *S*. Typhimurium DT56(v) isolates: 24/25 human and 35/38 garden bird-derived isolates, and clustered with groups 4 and 6. The profiles of PFGE groups 1 and 5 had a percentage similarity of 91%. These six groups (1–6) contained 97% (97/100) of all the isolates studied from both humans and birds, and had a percentage similarity >90%. Therefore it seems likely that the *Salmonella* isolates we analysed from both humans and birds are closely related.

**Table 1:** Pulsed-field gel electrophoresis group and host origin of *Salmonella* Typhimurium isolates.

Species	No. of isolates in PFGE group											
	1	2	3	4	5	6	7	8	9	10	11	12
**Human**	**20**	**0**	**0**	**1**	**24**	**0**	**2**	**1**	**0**	**1**	**1**	**0**
**Bird**	**9**	**2**	**1**	**0**	**35**	**1**	**0**	**0**	**1**	**0**	**0**	**1**
Bullfinch	0	0	1	0	1	0	0	0	0	0	0	0
Chaffinch	0	0	0	0	1	0	0	0	0	0	0	0
Goldfinch	0	0	0	0	1	0	0	0	0	0	0	0
Greenfinch	6	1	0	0	17	1	0	0	1	0	0	0
House sparrow	3	0	0	0	12	0	0	0	0	0	0	0
Siskin	0	1	0	0	3	0	0	0	0	0	0	1
**Phage type**	40	40	40	56v	56v	56v	40	40	56v	40	40	56v

### Temporal trends of garden bird and human isolates

**Figure 2:**
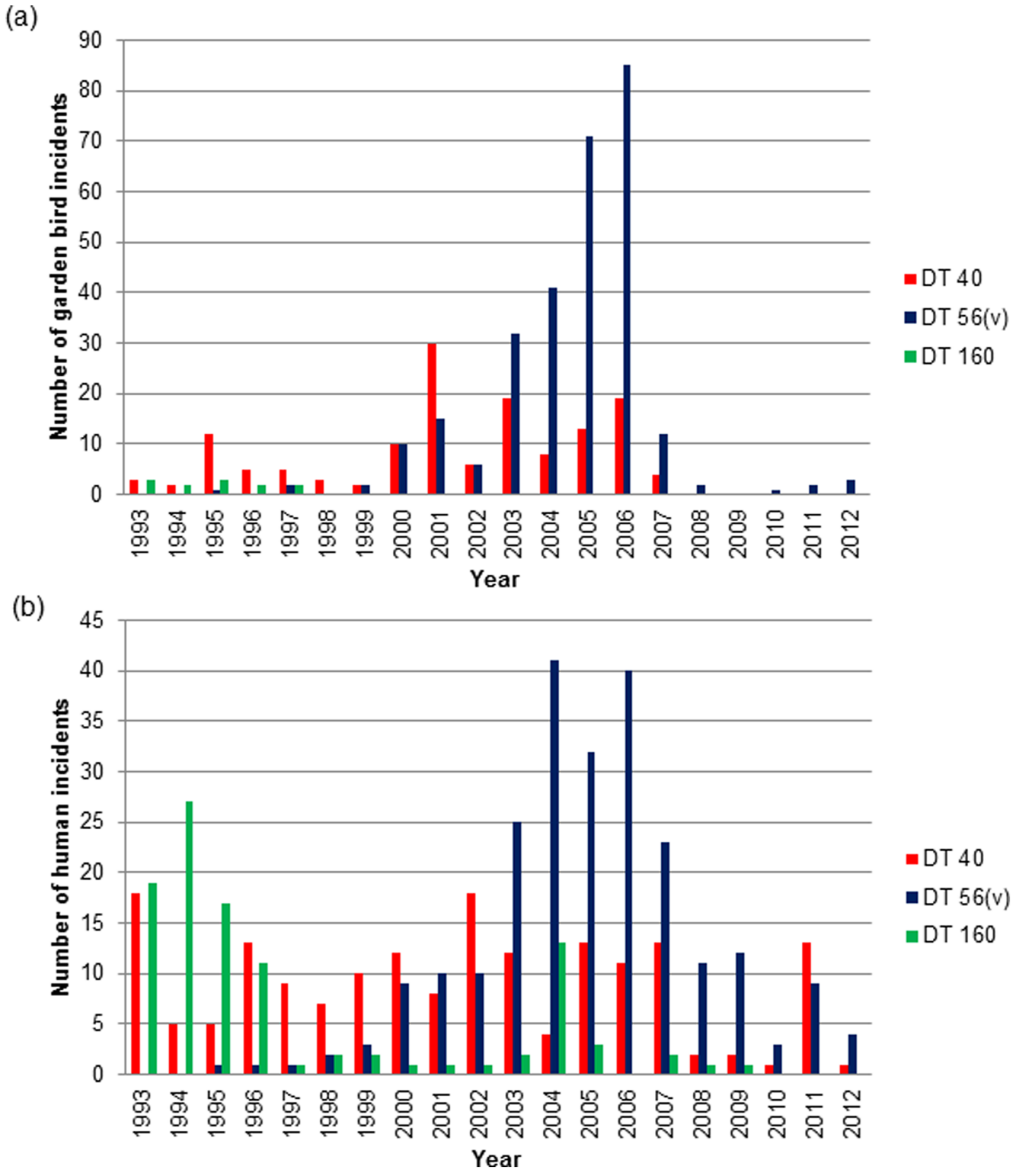
Number of garden bird and human incidents caused by *S*. Typhimurium phage types. (a) Number of garden bird incidents with *S*. Typhimurium DT40 (red), DT56(v) (blue) and DT160 (green), (b) Number of human incidents with *S*. Typhimurium DT40, DT56(v) and DT160 infection by year; 1993–2012.

Whilst garden bird incidents were confirmed in each year of the 20-year study with the exception of 2009, the frequency of *S*. Typhimurium phage types isolated from both birds and humans changed over the period (Figure 2). *S.* Typhimurium DT160 was only isolated from birds during the first five-year period of the study (1993–1997) and the majority of human isolates of this DT (72%, 75/104 incidents) were also from this period. *S.* Typhimurium DT40 was isolated throughout the study period until 2007. *S.* Typhimurium DT56(v) was found sporadically until 2000 and 2001, when the frequency of incidents increased considerably and it became the modal phage type from 2003–2012.

**Figure 3:**
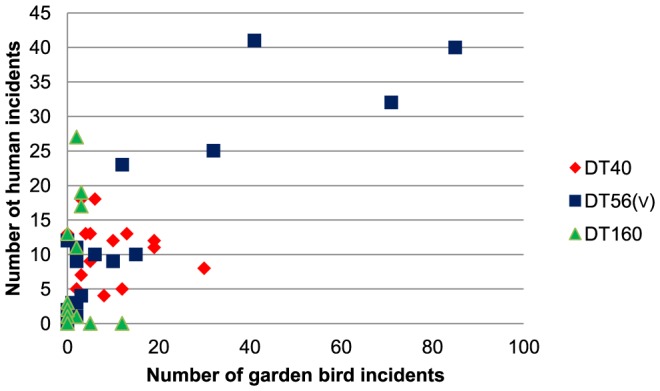
Number of garden bird incidents versus human incidents with *S*. Typhimurium infection by year, 1993–2012. Correlations for the individual phage types: DT56v r_18_ = 0.80, P<0.001; DT160 r_18_ = 0.59, P = 0.003 and DT40 r_18_ = 0.39, P = 0.046.

Annual trends were also observed in the human infection data for each of these *S.* Typhimurium phage types (Figure 2b). DT40 isolates were found throughout the study period, DT160 isolates were observed chiefly between 1993 and 1996 reducing to low numbers in later years (with a single anomalous peak in 2004) and DT56(v) incidents were identified with increasing frequency from 2000, becoming the modal phage type from 2003–2010 and in 2012. There was a significant positive association between the annual number of garden bird and human incidents across all phage types (r_18_ = 0.74, P<0.001) and for the individual phage types (Figure 3).

### Geographical distribution of garden bird and human isolates

**Figure 4:**
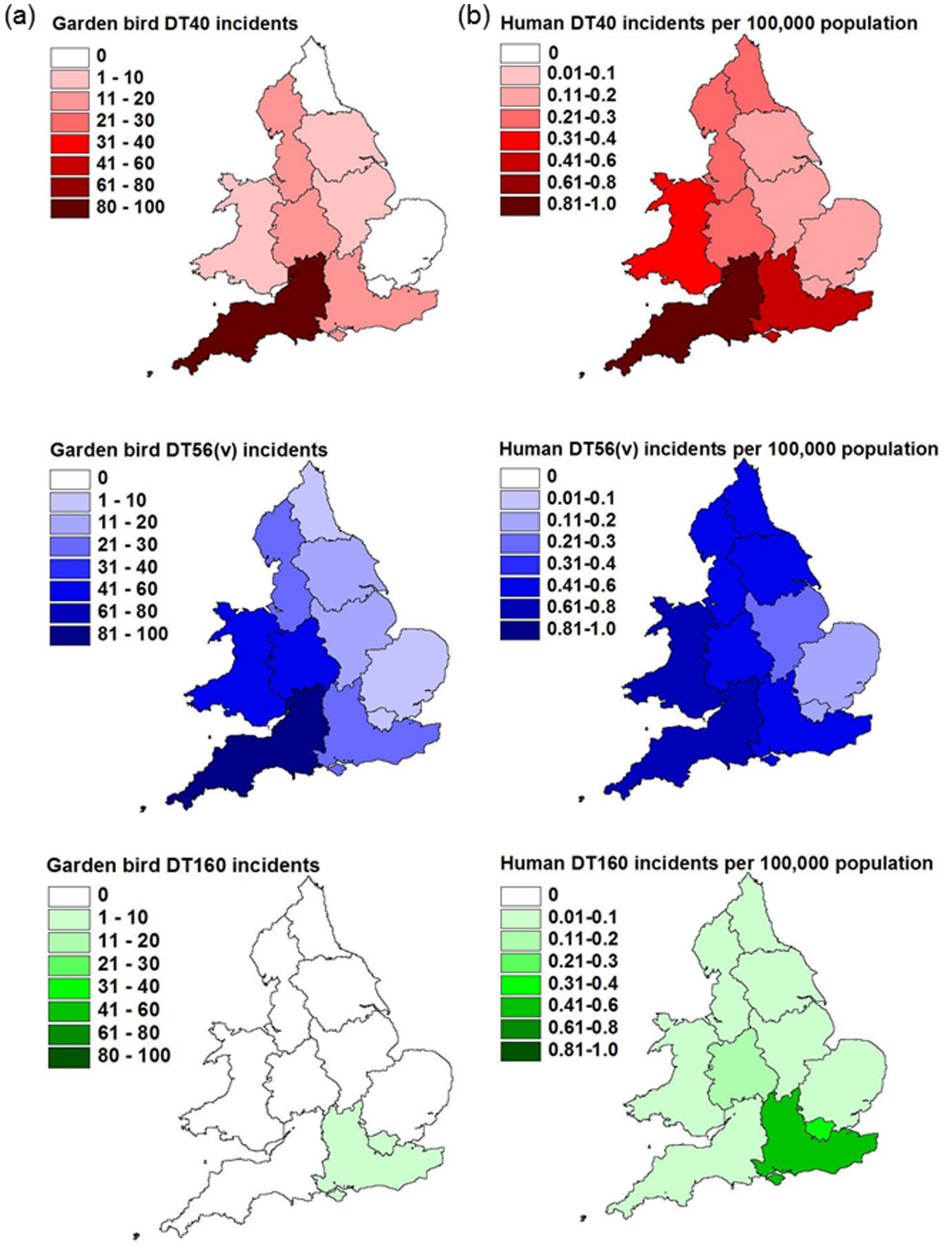
Distribution of *S.* Typhimurium DT40, DT56(v) and DT160 incidents, 1993–2012 in garden birds and humans. (a) Garden bird data expressed as total number of incidents by region (b) Human data expressed as number of incidents per 100,000 people by region, according to 2001 census data.

**Figure 5:**
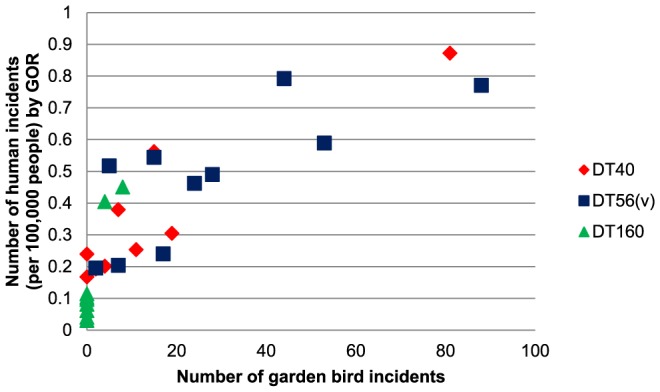
Number of garden bird incidents versus human incidents (per 100,000 people) with *S*. Typhimurium infection, 1993–2012. (a) Garden bird data expressed as total number of incidents by government office region (b) Human data expressed as number of incidents per 100,000 people by government office region, according to 2001 census data.

The spatial distribution of *S*. Typhimurium DT40, DT56(v) and DT160 human incidents was compared with that of the garden bird incidents using the GORs (Figure 4 and Figure 5). There was a significant positive association between the number of garden bird and human incidents (stratified by the human population census count), for each phage type; DT40 (r_8_ = 0.87, P = 0.001), DT56v (r_8_ = 0.73, P = 0.01) and DT160 (r_8_ = 0.70, P = 0.01).

Garden bird incidents due to phage type DT160 were clustered within South East England and London GORs; although human incidents occurred across England and Wales, the modal GOR regions were the same (Fig. 4).

The majority of garden bird incidents due to phage type DT40 occurred in South West England GOR, with the second most frequently affected GOR being the West Midlands. Incidents were reported in low numbers from across western and central England and Wales. In contrast, only six (4%; 6/141 incidents) garden bird DT40 incidents occurred in the four contiguous GORs along the eastern coast of England (North East, Yorkshire and Humber, East Midlands, East Anglia). Similarly, South West England was the modal GOR for human incidents which were reported from across Britain but with few from eastern England.

The spatial distribution of garden bird incidents due to phage type DT56(v) was similar to that of DT40; the South West, West Midlands and Wales were the three most frequently affected GORs, in rank order, whilst only 15% (44/285) of DT56(v) incidents were in the four contiguous GORs along the eastern coast of England (listed above). The GORs from which human incidents with DT56(v) occurred most frequently were the same as for wild birds (South West, West Midlands and Wales), with the lowest number of human incidents in East Anglia, London and the East Midlands.

## Discussion

Pulsed-field gel electrophoresis demonstrated a high degree of genetic similarity within and between *S.* Typhimurium DT40 and DT56(v) isolates derived from passerines and humans in this study. This finding strengthens previous studies [18], [35] which indicate that passerine-associated *S*. Typhimurium strains are host-adapted and that wild bird populations are the likely reservoir of these bacteria. In this study, the majority of garden bird and human-derived strains of matched phage type shared an identical PFGE banding profile; 94% (47/50) of the garden bird and 88% (44/50) of the human-derived isolates belonged to the two modal PFGE groups.

With this longitudinal study, we demonstrate a positive association between both the temporal and spatial patterns of reported garden bird and human incidents with matched *S.* Typhimurium phage types over a 20-year period. Taken together, the results of subtyping by phage typing and PFGE, and the similar temporal and spatial trends, provide evidence to support the hypothesis that wild birds are the primary source of human infection with these *S*. Typhimurium phage types in Britain.

Direct comparison of the number of garden bird incidents diagnosed between years is problematic in this study because of the opportunistic nature of the surveillance and the inconsistent (and unknown) observer effort between years. Nevertheless this study extends significantly previous research [8], [18], highlighting the dynamism of passerine salmonellosis, both in terms of the number of incidents by year and the succession of predominant phage types. Published longitudinal monitoring of cattle populations in England and Germany also revealed changes in the relative frequency of isolation of *S*. Typhimurium phage types over time [15]. The mechanism for phage type succession in captive and wild animal populations and the risks for human populations is therefore an important issue which requires further investigation.

For wild birds in Britain the spatial distribution of passerine salmonellosis incidents with DT40 and DT56(v) was previously described [8], [18]. The relative abundances of house sparrow and greenfinch populations (the two species most commonly diagnosed with salmonellosis) across Britain, however, do not mirror the spatial DT patterns. Other proposed factors to explain the DT spatial patterns observed include habitat, climate, agricultural practices and wild bird movements [8].

A limitation of this study is the crude geographical resolution available for the human incidents. Since patient confidentiality limited data access, the referring hospital was the best available proxy for location. Variation in distance from home to the investigating medical facility, and contraction of infection when away from home with subsequent medical investigation following the patient's return, are potential sources of error. This necessitated exploration of the spatial data by broad region; nevertheless, the garden bird and human data spatial trends were remarkably similar.

Human beings might become infected with garden bird *S*. Typhimurium phage types via direct or indirect routes of exposure. Handling of sick and dead garden birds has been identified as a risk factor for human infection with *Salmonella* [36], [37]. Handling clinically healthy birds excreting *Salmonella* spp. also has the potential to result in infection, with licensed bird ringers and other ornithologists being a demographic group at risk of exposure [38]. Whilst little information is available on the rates of subclinical carriage of these *S.* Typhimurium strains in apparently healthy garden birds in Britain, available data from migratory and resident passerine populations in Europe [39]–[41] indicate that the risk of exposure to *Salmonella* associated with handling healthy passerines is likely to be low. For example, only 2% (40/1990) of apparently healthy passerines caught by bird ringers at feeding stations in Norway, during the winters 1998–2000, had *Salmonella* spp. isolated from cloacal swabs [41]. Carrier species are chiefly those which most frequently succumb to clinical salmonellosis [41].

Human infection could also result through indirect routes of exposure, such as cleaning contaminated bird feeders in the kitchen in close proximity to human food preparation areas or contact with bridge species (livestock, wildlife, companion animals). Sporadic infection of livestock and companion animals with garden bird-associated *S*. Typhimurium phage types has been demonstrated in Britain [42]. The domestic cat, for example, could act as a bridge species following infection through predation or scavenging of infected birds [4], [23]. Healthy domestic cats, however, are likely to present a low risk of *Salmonella* excretion; rectal swabs from 278 healthy domestic cats (with a range of outdoor access dependent on the owner) were screened in Belgium and *Salmonella* sp. (*Salmonella* Enteriditis phage type 21) was isolated from a single cat [43].

Indirect transmission of infection to people also might occur via contact with environmental sources (e.g. garden soil) contaminated with wild bird faeces. Gardening and outdoor recreation activities might increase the risk of exposure. Infants and children may be exposed during outdoor play, particularly from contaminated soil in the vicinity of garden bird feeding stations. In a Norwegian study, *Salmonella* spp. was isolated from waste food and soil from two sites with a history of garden bird salmonellosis incidents, indicating environmental persistence of the bacterium at infected sites [41]. Numerous factors influence *Salmonella* spp. persistence in the environment, including temperature, moisture, soil type and UV exposure. Experimental studies have provided evidence for long-term environmental persistence; for example *Salmonella* was found to persist for 96 days at 8°C in garden soil [44], 180 days in manure-contaminated soil during simulated summer-winter exposure [45], and 280 days in urban garden soil [46].

Unfortunately, detailed clinical histories were not available for the patients in this study; consequently risk factor analyses which might further inform routes of transmission could not be performed. An outbreak of *S*. Typhimurium DT160 infection in human beings that occurred when this strain of the bacterium first emerged as a cause of garden bird mortality in New Zealand was investigated and the authors' determined that contact with dead wild birds, contact with people with gastrointestinal disease and consumption of fast food were significantly associated with the risk of infection [37].

In a prospective case control study of human infections with *S*. Typhimurium O:4,12 in Norway, patients were questioned about the two-week period preceding the onset of their clinical signs [36]. Detailed questionnaires explored multiple potential risk factors and concluded that drinking untreated water, direct contact with wild birds or their droppings and ingestion of snow, sand or soil were related to an increased risk of infection. Of the ten respondents who reported a history of direct contact with wild birds or their droppings, six had cleaned a bird feeder or removed bird faeces and four had touched a dead bird or nursed a sick bird. No increase in risk was observed for people who fed garden birds, or for other family members who shared a household where bird feeding was practiced. Also, there was no increased risk for people in contact with other wild, captive or domestic animals [36].

Infants were the modal age group confirmed with human infection for each of the garden bird-associated *S*. Typhimurium phage types in the current study (242/518; 47% overall), which mirrors the general trend reported by the PHE for all *S*. Typhimurium phage types. The average annual number of cases of *Salmonella* infections in humans (including all *Salmonella* spp. and serovars), 2000–2010, reported by PHE for England and Wales was greatest for babies (<1 year old; 96.7 (35% of all *Salmonella*) per 100,000 individuals) and then infants (1–4 years old; 58.5 (21%) per 100,000 individuals) and lower in the adult age groups (15–44 years old, 24.0 (9%) per 100,000 individuals; 45–64 years old, 21.8 (8%) per 100,000 individuals; 65–74 years old, 17.0 (6%) per 100,000 individuals; >75 years old, 12.4 (4%) per 100,000 individuals) [47]. Similar trends have been described in the demography of human infection with garden bird-associated *S*. Typhimurium strains from other studies [21], [36], [37]. In Norway, 43% of the 153 sporadic human cases of infection with *S*. Typhimurium O:4,12 (over the period 1966–1996, excluding cases during an outbreak in 1987), a strain-associated with wild passerines, were less than five years old [36]. Similarly, the Scottish Salmonella Reference Unit found that, between 2001 and 2007, 38% of 47 *S*. Typhimurium DT40 isolates and 52% of 29 DT56(v) isolates in human beings were from children <5 years old [21]. In New Zealand, the median age of patients with *S*. Typhimurium DT160 infection was 8 years (range 4 months – 90 years) [37].

Evidence suggests that infants and elderly people are more susceptible than adults to illness following challenge with low numbers of *Salmonella* sp. bacteria [48]. Infants are more likely than adults to be immunologically naïve to salmonella infection [49] and they might be more likely to be presented for clinical investigation of gastroenteritis than older patients. In addition, infants and children are likely to have poorer levels of personal hygiene which, coupled with play in outdoor environments, is likely to increase their exposure to environmental *Salmonella* spp. bacteria than older demographic groups.

Antimicrobial susceptibility studies of *S*. Typhimurium isolates from garden birds in the north of England show minimal antibiotic resistance, indicating that they do not represent an important source of resistant infection to the human population [17]. Hughes et al. [17] also determined that the 32 garden bird-derived isolates they examined lacked the translocated effector protein (*sopE*) gene which has been frequently associated with some epidemic strains of *S*. Typhimurium in humans and livestock [50], indicating that wild birds might not pose a high zoonotic risk. The human *S*. Typhimurium infections in the current study were all considered sporadic infections and no large outbreaks of disease were recorded with these strains of the bacterium.

Whilst this study supports the hypothesis that garden birds act as the primary reservoir of human infection with garden bird-associated *S*. Typhimurium phage types in England and Wales, these findings must be viewed in context. The PHE confirmed a total of 147,495 human infections with all *Salmonella* spp., including 20,785 human infections with *S.* Typhimurium, from 2000–2010 across England and Wales [47]. In this study, we identified a total of 337 human infections with garden bird-associated *S*. Typhimurium DT40, DT56(v) and DT160 isolates, 2000–2010, which accounted for only 0.2% of all the *Salmonella* spp. infections diagnosed and only 1.6% of the *S*. Typhimurium isolates obtained during this 11-year period. The number of human incidents of salmonellosis is likely to be under-reported, however, as people with mild cases of gastroenteritis may not present to the medical community for further investigation and, if they do, samples might not be submitted for bacteriological analysis.

The importance of garden birds as a potential reservoir of *S.* Typhimurium infection in Britain should be kept in perspective, but members of the public who feed garden birds should be aware that wild birds can carry zoonotic pathogens. Sensible routine hygiene precautions, such as avoiding direct contact with bird faeces and carcasses and washing hands after feeding birds, are recommended to reduce the risk of exposure. Any public health risk can be further mitigated by raising the public, veterinary and medical communities' awareness of salmonellosis as a cause of garden bird disease, the risk of salmonellosis in domestic cats subsequent to passerine predation, measures for control where disease outbreaks occur and routine best practice for the feeding of garden birds.

Further characterisation, using whole genome sequencing, of *S*. Typhimurium strains associated with garden birds and matched phage types from humans is required to better understand their ancestry and accessory gene content and to further explore the hypothesis that garden birds are the primary reservoir hosts of these pathogens.
